# Comparison between manual and automated methods for the isolation of mononuclear cells and mesenchymal stem cells using ficoll: efficacy and reproducibility

**DOI:** 10.3389/fcell.2025.1556697

**Published:** 2025-03-21

**Authors:** Ester Moñivas, Concepción Aguayo, Silvia de la Calle, Paula Martínez, Nieves Repollés, Mercedes Zurita

**Affiliations:** ^1^ Instituto de Investigación Sanitaria Puerta de Hierro - Segovia de Arana, Hospital Universitario Puerta de Hierro Majadahonda, Majadahonda, Spain; ^2^ Cell Therapy Unit, Puerta de Hierro University Hospital Majadahonda, Madrid, Madrid, Spain

**Keywords:** bone marrow, mononuclear cells, mesenchymal stem cells, ficoll, sepax

## Abstract

Mesenchymal stem cells (MSCs), hold immense therapeutic promise in Advanced Therapy Medicinal Products (ATMPs) due to their multipotent nature, immunomodulatory properties, and anti-inflammatory effects. However, a significant challenge lies in obtaining sufficient quantities of MSCs for therapeutic applications, necessitating *ex-vivo* culture of Mononuclear Cells (MNCs) isolated from source tissues like bone marrow. This study compares the efficacy of MNC isolation using manual and automated methods, specifically evaluating the Sepax system, and investigates whether the isolation method impacts MSCs yield. Seventeen bone marrow samples were processed using both methods, with subsequent analysis of MNC and colony-forming unit (CFU) counts, MSCs differentiation potential, and phenotypic characterization. While the Sepax system demonstrated slightly higher MNC yields, no significant differences were observed in CFU formation or MSCs characteristics compared to manual isolation. These findings underscore the importance of critically evaluating isolation methods to ensure both efficiency and quality in therapeutic applications.

## Introduction

Mesenchymal Stem Cells (MSCs) are multipotent stem cells, adherent *in vitro* culture and with a fibroblast-like morphology, capable of differentiating into cells of mesodermal origin such as osteocytes, chondrocytes and adipocytes, which makes them a promising option for therapeutic applications in Advanced Therapy Medicinal Products (ATMPs). These cells can be obtained from various sources, including adipose tissue, umbilical cord, and bone marrow (Dominici et al., 2006). The latter has been established as the most commonly used source in current literature due to its abundance and accessibility (Thirumala et al., 2009; Gudleviciene et al., 2014). In addition to their differentiation capacity, MSCs also exhibit immunomodulatory and anti-inflammatory properties, making them ideal candidates for the treatment of various diseases and disorders (Najar et al., 2016).

However, a significant limitation in the clinical application of MSCs is the low quantity of cells obtained from source tissues, necessitating *ex-vivo* culture to obtain a sufficient quantity for the formulation of ATMPs. For this purpose, efficient isolation of Mononuclear Cells (MNCs) present in the bone marrow is crucial, a fundamental step in the MSC procurement process (Chu et al., 2020; Nasef et al., 2007). Isolation of MNCs can be achieved through density gradient cell separation, using media such as Ficoll-Paque PLUS (Cytiva). This process can be performed either automatically using specialized equipment such as the Sepax S-100 (Biosafe, Eysins, Switzerland), or manually (Aktas et al., 2008; Kaur et al., 2017).

In this study, we address the comparison between the isolation of MNCs using the density gradient technique, both automated with the Sepax S-100 equipment and manually. Additionally, we will investigate whether the method that best isolates MNCs in the bone marrow correlates with greater efficiency in obtaining MSCs. It is important to highlight that all studies, from the isolation of MNCs to the evaluation of efficiency in obtaining MSCs, were conducted in a cleanroom following strict Good Manufacturing Practice (GMP) regulations, aiming to provide more reliable and relevant data in optimizing ATMP manufacturing processes. The operators involved in the activities carried out in the present study possess the necessary training, experience, and qualifications for the manufacturing of advanced therapy medicinal products within a controlled environment under. They receive training in Good Manufacturing Practices, including the proper handling of cleanrooms, environmental control, and aseptic techniques. Additionally, they have the required knowledge of current regulations and participate in continuous training and periodic certifications to ensure competence and compliance with current standards. This rigorous methodology contributes to consolidating the validity and clinical applicability of the results obtained in this study (Aktas et al., 2010).

## Materials and methods

### Number of samples studied

A total of 17 samples were included in this study. The samples were obtained from the bone marrow aspiration of patients scheduled for treatment at the Cellular Therapy Unit with the autologous Advanced Therapy Medicinal Product NC1, authorized under Authorization No. 83976 by the Spanish Agency for Medicines and Medical Devices (AEMPS). The cohort consisted of patients with chronic traumatic spinal cord injury, aged between 18 and 65 years, including 13 men and 4 women. Patient selection was conducted by a multidisciplinary committee at Puerta de Hierro University Hospital, based on predefined clinical criteria to initiate the manufacturing process of the medicinal product NC1. All patients were fully informed about the study and provided written informed consent. The bone marrow samples used in this study were further processed and administered to the selected patients as part of the NC1 treatment.

### Bone marrow aspiration

Bone marrow aspiration was performed using 20 mL syringes containing 250 U/mL of sodium heparin (Rovi) and an antibiotic-antimycotic solution (GibcoBRL). This procedure was carried out in the operating room by puncturing the iliac crest of patients under local anesthesia.

### Isolation of MNCs using the manual method

For the isolation of MNCs using the manual method, a 100 mL sample of undiluted bone marrow was processed. Five 50 mL tubes (Corning) were used for manual processing. In each 50 mL tube, 100 mL of Ficoll (Ficoll-Paque PLUS, Cytiva) was evenly distributed. Subsequently, the transfer bag containing the bone marrow was carefully dispensed into the 50 mL tubes, adding 20 mL of the sample to each one. Density gradient centrifugation was carried out for 30 min at 300g and 21°C. After centrifugation, the MNC phase was collected and washed with minimal essential medium (a-MEM; Bio-Whittaker) supplemented with 20% FBS (Bio-Whittaker), 10 mmol of glutamine (GibcoBRL), and 1% antibiotic-antimycotic solution (GibcoBRL) before a new centrifugation for 10 min at 1,250 rpm and 21°C. Finally, the resulting pellet was unified and resuspended in a volume of 50 mL of wash medium.

### Isolation of MNCs using sepax

For the isolation of MNCs using the Sepax system a 100 mL sample of undiluted bone marrow was processed MNCs were separated using a density gradient employing the automated cell processing system Sepax (BioSafe) with the single-use kit DGBS/Ficoll CS-900 (Biosafe). This method is based on density gradient centrifugation using specific media.

Initially, the bone marrow collection bag was connected to the kit’s input port, along with a wash solution bag containing 500 mL of minimal essential medium (a-MEM; Bio-Whittaker) supplemented with 20% FBS (Bio-Whittaker), 10 mmol of glutamine (GibcoBRL), and 1% antibiotic-antimycotic solution (GibcoBRL). Additionally, the kit’s waste/Ficoll bag was filled with 100 mL of lymphocyte separation medium (Ficoll-Paque PLUS, Cytiva). Upon completion of the sample processing, the isolated MNCs were recovered in the 150 mL transfer bag provided in the kit, in a volume of 50 mL of wash medium.

### Cell counting

Cell counting was performed using the Sysmex XN-20 equipment (Sysmex Corporation), a hematology analyzer capable of examining different cellular components of blood and body fluids. The XN-20 analyzer enhances white blood cell (WBC) signaling, which, combined with Sysmex parameters, improves the efficiency of automated MNCs analysis.

### Obtaining MSCs: comparison between manual method and sepax automated system

For MSCs procurement, MNCs obtained using both the manual method and the Sepax automated system were separately cultured. MNCs were seeded at a concentration of 160,000 cells/cm^2^ in 175 cm^2^ culture flasks (Thermo Fisher Scientific), using minimal essential medium (a-MEM; Bio-Whittaker) supplemented with 20% fetal bovine serum (FBS) (Bio-Whittaker), 10 mmol of glutamine (GibcoBRL), and a 1% antibiotic-antimycotic solution (GibcoBRL). The seeded cells were cultured in a CO_2_ incubator under strictly controlled conditions,at 37°C with 5% CO_2_ humidified to 100%. These conditions are essential to maintain an appropriate physiological pH in the culture medium and to promote optimal cell growth. After 24 h, adherent cells (MSCs) were detached using trypsin/ethylenediaminetetraacetic acid (EDTA) solution (BioWhittaker-Lonza) for 15 min at 37°C. Trypsin neutralization and subsequent washing were performed with minimal essential medium (a-MEM; Bio-Whittaker) supplemented with 20% FBS (Bio-Whittaker), 10 mmol of glutamine (GibcoBRL), and a 1% antibiotic-antimycotic solution (GibcoBRL), centrifuging at 1,250 rpm for 10 min. Subsequently, MSCs were resuspended in 2 mL of minimal essential medium (a-MEM; Bio-Whittaker) supplemented with 20% FBS (Bio-Whittaker) and 10 mmol of glutamine (GibcoBRL), and MSCs counting was conducted using the Sysmex XN-20 equipment (Sysmex Corporation).

### Colony-forming unit assay

MSCs were seeded in 60 cm^2^ Petri dishes (Thermo Fisher Scientific) at a density of 215 MSCs/dish for each condition. After 14 days in culture at 37°C with 5% CO_2_ humidified to 100%, the culture medium was removed, and cells were fixed for 30 min with 4% paraformaldehyde. Subsequently, cells were washed with PBS 1X and stained with 0.5% cresyl violet for 10 min.

### *In vitro* differentiation studies

#### Adipogenic differentiation

Adipogenic differentiation was carried out using the MSC Differentiation Bullet Kit Adipogenic (Lonza). The culture system includes a basal medium and the supplements required for the maintenance and induction of hMSC adipogenic differentiation. According to the technical datasheet, the hMSC Adipogenic Induction SQ Kit is composed of Indomethacin, IBMX, recombinant human insulin (0.1%), dexamethasone, fetal bovine serum, L-glutamine, and GA-1000, while the MSC Adipogenic Maintenance SQ Kit is supplemented with recombinant human insulin (0.1%), fetal bovine serum, L-glutamine, and GA-1000. Both media were prepared immediately before use.

MSCs isolated at a density of 2.1 × 10^4^ cells per cm^2^ were seeded into a 60 cm^2^ culture dish (Thermo Fisher Scientific) with 0.2 or 0.3 mL of medium per cm^2^ of surface area and incubated at 37°C with 5% CO_2_ humidified to 100%. The medium was replaced every 3 days until the cultures reached 100% confluence, approximately 14 days with no time differences between both methods. Three cycles of induction/maintenance were performed with the respective media, followed by 7 days of maintenance in the maintenance medium. The evaluation of results was conducted by staining with 0.35% Oil Red (Sigma-Aldrich) in distilled water for 50 min with agitation at room temperature, followed by microscopic observation for assessment.

#### Osteogenic differentiation

Osteogenic differentiation was performed using the Human Mesenchymal Stem Cell Osteogenic Differentiation Medium Bullet Kit™ (Lonza). This culture system includes the required basal medium and supplements for MSC osteogenic differentiation. According to the technical datasheet, the basal medium (MSCDM-Ost Basal Medium) is supplemented with the following induction components: dexamethasone, β-glycerophosphate, ascorbate, penicillin-streptomycin (10k/10k), fetal bovine serum, and L-glutamine.

MSCs isolated at a density of 3.1 × 10^3^ cells per cm^2^ were seeded into a 60 cm^2^ culture dish (Thermo Fisher Scientific) with 0.2 or 0.3 mL of induction medium per cm^2^ of surface area and incubated at 37°C with 5% CO_2_ humidified to 100%. The medium was replaced every 3 days with fresh medium until reaching 100% cellular confluence, approximately 14 days with no time differences between both methods. The evaluation of results was conducted by staining with Alkaline Phosphatase (Sigma-Aldrich) for 15 min in darkness at room temperature, followed by microscopic observation for assessment.

#### Chondrogenic differentiation

Chondrogenic differentiation was carried out using the Human Mesenchymal Stem Cell Chondrogenic Differentiation Medium Bullet Kit™ (Lonza), which includes both the basal medium and the necessary supplements: ITS, sodium pyruvate, proline, dexamethasone, ascorbate, L-glutamine, and GA-1000, with the addition of TGF-β3 for chondrogenic differentiation, as specified in the product datasheet.

Chondrogenesis induction was performed using the micropellet formation technique with MSCs isolated at a cellular density of 2.5 × 10^5^ cells in chondrogenic medium. The cells were centrifuged at 300 *g* for 10 min, and the resulting pellet was cultured in chondrogenic medium at 37°C with 5% CO_2_ humidified to 100%. The chondrogenic medium, supplemented with TGF-β3, was replaced every 3 days over a 3-week period. Subsequently, chondrogenic differentiation of the micropellets was evaluated by staining with 0.1% Safranin O for 3 min at room temperature. Finally, the cells were observed under an inverted microscope.

### Inmunophenotypic characterization of MSCs

For the phenotypic characterization of MSCs, monoclonal antibodies conjugated with different fluorochromes (Fluorescein [FITC], Phycoerythrin [PE], and Alexa-647 [AL-647]) were used to analyze a variety of cell surface markers. The specific antibodies used included CD105 FITC (R&D Systems), CD90 AL-647 (AbD Serotec, OX5 1GE), HLA Class I FITC (Cytognos), CD73 PE (BD Bioscience), CD166 PE (R&D Systems), CD34 PE (BD Bioscience), HLA Class II PE (Cytognos), CD80 AL-647 (AbD Serotec), CD45 FITC (Cytognos), and CD31 FITC (Cytognos).

To ensure the specificity of the monoclonal antibodies, appropriate isotype controls for FITC and PE (Cytognos), and AL-647 (AbD Serotec) were used. The labeled cells were analyzed using a FC500 MPL Cytomics flow cytometer (Beckman Coulter) with MXP software (Beckman Coulter). Non-viable cells were excluded using the LIVE&DEAD reagent (Invitrogen), and the collected data were analyzed with CXP Analysis software, version 2.1 (Beckman Coulter).

### Ki67 immunohistochemistry

Isolated MSCs are seeded at a concentration of 2,000/cm^2^ onto culture slides and incubated at 37°C with 5% CO_2_ humidified to 100%. After 24 h, the slides are fixed in 4% paraformaldehyde for 30 min and washed with distilled water for 10 min, followed by three washes of 5 min each with PBS 1X (Thermo Fisher Scientific). Subsequently, the sections were treated with citrate buffer (pH 6.0) for 20 min in a heat cooker for antigen unmasking, followed by three washes with PBS 1X (Thermo Fisher Scientific). Next, the slides were incubated with 30% hydrogen peroxide to block endogenous peroxidase activity and washed with PBS 1X (Thermo Fisher Scientific). Afterwards, the slides were incubated for 1 h at room temperature with non-immune serum blocking solution to prevent nonspecific binding. Then, the primary Rabbit Anti-Human Ki-67 Monoclonal Antibody (master diagnostica) was added and incubated at 4°C overnight in a humid chamber. After the primary antibody incubation time, the slides were washed with PBS 1X and biotinylated secondary antibody (Master polymer plus HRP) was added at a dilution of 1/200 in blocking solution, incubated for 1 h at room temperature in a humid chamber. To reveal the secondary antibody, they were incubated with an ABC complex (streptavidin) for 1 h at room temperature in a humid chamber. After the incubation time, to develop color reaction, a diaminobenzidine chromogen (master diagnostic developing Kit) was added for a maximum of 15 min at room temperature. Contrast staining was performed with hematoxylin for 3 min, and preparations were mounted with EUKIT. Marker expression will be carried out using a ki67 labeling index expressed as a percentage. To do this, we will proceed to count the stained cells compared to the total cells.

### Statistical analysis

A combination of statistical tools was used to examine differences in the isolation of MNCs and MSCs between manual ficollization method and the SEPAX automatic system. This evaluation included analysis using the Bland & Altman plot, as well as calculation of the correlation and concordance coefficient by Lin (rho_c). Additionally, Student’s t-test was performed to determine differences found in Colony-Forming Units counts and the percentage of Ki67 between MSCs obtained manually and using the SEPAX automatic system.

To further ensure the robustness of the conclusions, a *post hoc* power analysis was conducted. The analysis evaluated the observed concordance correlation coefficients (ρc) for MNC and MSCs to determine if the sample size (n = 17) was sufficient. Using the observed values (ρc = 0.749 for MNC and ρc = 0.849 for MSC), Cohen’s d effect sizes were calculated (d = 4.66 and d = 6.62, respectively). The statistical power for both variables was found to be 1.0 (100%) at a significance level of α = 0.05. These results demonstrate that the sample size provided sufficient power to detect meaningful concordance, supporting the validity of the findings

## Results

### MNC recovery. Comparative analysis of ficollization between sepax and manual methods

A comparative analysis was conducted between samples isolated with the SEPAX system and samples processed manually, evaluating the agreement between both procedures using the Lin concordance correlation coefficient (rho_c). The median (p50) along with the 25th and 75th percentiles for samples treated with SEPAX and manually are presented in Table 1. Additionally, the Lin concordance correlation coefficient (rho_c) with its 95% confidence interval is shown. Subsequently, the Bland & Altman plot was used to visualize the agreement between the two methods.

**TABLE 1:** Agreement between MNC and MSCs. Recovery using manual method or sepax analyzed by bland-altman plot.

Cell Type	Mean difference	Standard deviation	Lower 95% limit	Upper 95% limit
MNC	2.591	5.411	−8.016	13.197
MSCs	−0.049	0.293	−0.624	0.526

The results showed that the median (p50) WBCs count was slightly higher in samples treated with the SEPAX system (14.01 (25th percentile: 7.64, 75th percentile: 20.64)) compared to the manual method (12.76 (25th percentile: 7.27, 75th percentile: 17.21)). However, the Bland & Altman plot displayed an average difference of 2.591, with a standard deviation of 5.411. This indicates that, on average, the SEPAX system tends to provide higher counts than the manual method.

The Lin concordance correlation coefficient (rho_c) was 0.749 (95% CI: 0.551-0.948), suggesting good agreement between the two methods. However, upon observing the Bland & Altman plot, the 95% agreement limits were −8.0162 to 13.197, showing considerable variability in differences between the methods, with some cases exhibiting significant discrepancies of more than 10 units.

The wide confidence intervals noted in the Bland-Altman analysis for MNCs (−8.016–13.197) primarily reflect inherent biological variability in the bone marrow samples, which is expected when working with human-derived tissues. Despite this, the Lin concordance correlation coefficient (ρ_c = 0.749) demonstrates good agreement between the manual and automated methods, supporting the reliability of both approaches.

### MSCs recovery. Comparison between sepax and manual methods

In this study, the quantity of isolated MSCs using the automatic SEPAX equipment was compared with manual isolation. The results are presented in Table 2. We observed that the median levels of MSCs were slightly lower in samples treated with the SEPAX system (0.66 (25th percentile: 0.47, 75th percentile: 1.03)) compared to the manual method (0.69 (25th percentile: 0.64, 75th percentile: 1.22)). The range of MSCs isolated using the automatic SEPAX method was between 0.74% and 5.88%, while the manual method showed a range of 1.08%–7.65%. However, when evaluating the agreement between both methods using the Lin concordance correlation coefficient (rho_c), we found a value of 0.849 (95% CI: 0.709-0.988), indicating substantial agreement between the two methods.

**TABLE 2:** MNC and MSCs recovery between sepax and manual methods.

Parameter	MNC sepax	MNC manual	MSCs sepax	MSCs MSC manual
n	17	17	17	17
p25	7.64	7.27	0.47	0.64
p50	14.01	12.76	0.66	0.69
p75	20.64	17.21	1.03	1.22

The Bland & Altman analysis revealed an average difference between the methods of −0.049, suggesting good agreement. However, it was observed that two patients were outside the 95% agreement limits, these deviations may be attributed to biological variability. The results suggest satisfactory agreement between the SEPAX and manual methods in measuring MSCs levels. Although most observations fall within the agreement limits, it is important to note discrepancies in some individual cases.

### Colonial forming units

**FIGURE 1:**
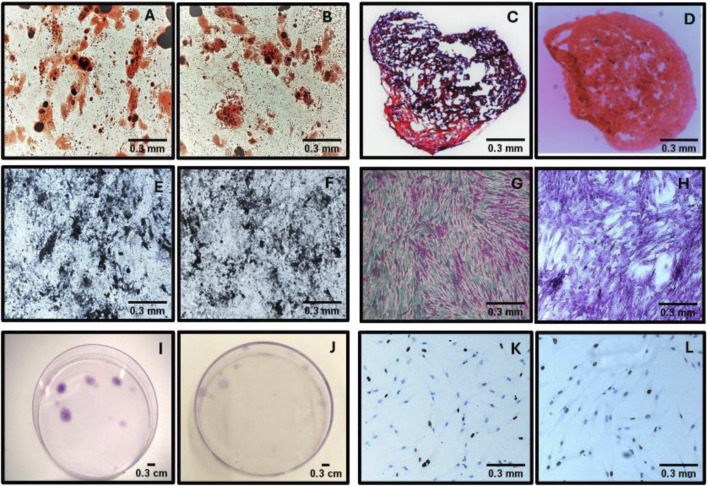
Comparative assessment of MSCs isolated using manual and automated methods. **(A–B)** Adipogenic differentiation potential in MSCs isolated manually **(A)** and through automation **(B)**. **(C–D)** Chondrogenic differentiation potential in MSCs obtained via manual **(C)** and automated **(D)** isolation. **(E–F)** Osteogenic differentiation potential in MSCs from manual **(E)** and automated **(F)** methods. **(G–H)** Colony formation capacity assessed microscopically in MSCs cultured using manual **(G)** and automated **(H)** methods. **(I–J)** Macroscopic observation of MSC colonies in culture plates under manual **(I)** and automated **(J)** conditions. **(K-L)** Proliferative activity assessed by Ki67 immunostaining in MSCs obtained manually **(K)** and via automation **(L)**.

The mean number of Colonial Forming Units (CFU-F) obtained through the CFU-F assay was 5.4 ± 5.48 for manually isolated MSCs and 4.99 ± 5.68 for MSCs isolated using the automated Sepax system. A statistical analysis was performed using the Student’s t-test to assess if there were significant differences between the two methods. With the analyzed data, we obtained a t-value of −0.51, p = 0.62 (α = 0.05), indicating that the two methods could be considered equivalent in terms of their efficacy in colony formation. A representative image of the colony-forming units is shown in Figure 1.

### Differentiation of MSCs

The differentiation potential of MSCs, isolated both manually and automatically with the Sepax system, was confirmed by demonstrating their adipogenic, chondrogenic, and osteogenic differentiation *in vitro* (Figure 1).

### Proliferative activity of adherent cells by Ki67 marker study

The expression of the Ki67 marker in isolated MSCs was evaluated by immunohistochemistry (Figure 1). A mean of 52.96 ± 27.35 was obtained for manually isolated MSCs and 52.02 ± 27.30 for MSCs isolated using the automated Sepax system. A statistical analysis was performed using the Student’s t-test to establish if there were significant differences between the two isolation methods. The t-value was equal to −0.52 with a p-value of 0.61 (significance level α = 0.05). These results suggest that there are no differences in the expression of the Ki67 marker when comparing the results obtained between manually isolated MSCs and those isolated using the automated Sepax system.

### Inmunophenotypic characterization of MSCs

The immunophenotyping results of MSCs isolated both manually and automatically showed no significant differences in their phenotypic profiles between the two isolation methods. Flow cytometry analysis confirmed that the expression levels of key surface markers were consistent across both methodologies, indicating the robustness and reproducibility of the isolation techniques. For the manually isolated MSCs, the percentage expression of surface markers was as follows: CD105 (65.43% ± 4.23), CD34 (2.01% ± 0.87), CD90 (32.98% ± 3.54), HLA-II (66.18% ± 5.12), HLA-I (89.40% ± 3.98), CD80 (9.53% ± 2.14), CD45 (70.18% ± 4.76), CD73 (16.78% ± 2.87), CD31 (26.58% ± 3.21), CD166 (72.52% ± 4.11), and CD14 (61.84% ± 3.97). Similarly, for MSCs isolated using the automated method, the expression levels were as follows: CD105 (66.80% ± 4.11), CD34 (1.68% ± 0.73), CD90 (29.32% ± 3.29), HLA-II (67.20% ± 4.98), HLA-I (90.53% ± 3.76), CD80 (8.05% ± 2.06), CD45 (74.17% ± 4.54), CD73 (13.74% ± 2.63), CD31 (24.96% ± 3.09), CD166 (76.03% ± 4.23), and CD14 (65.38% ± 3.82).

**FIGURE 2:**
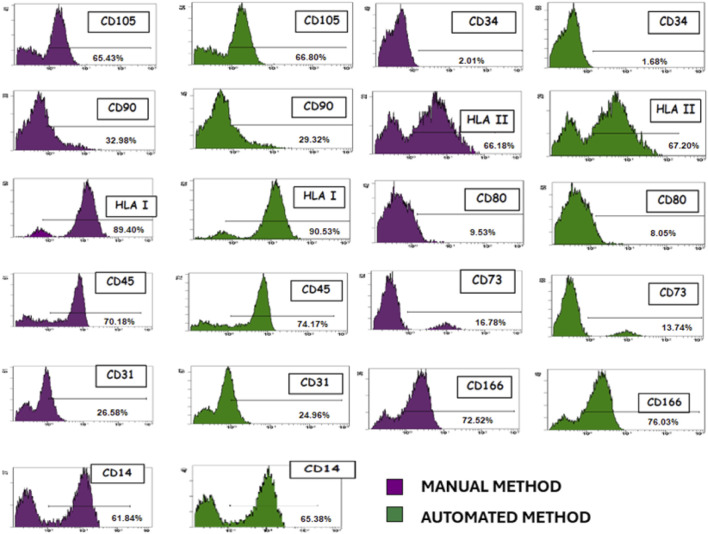
Representative immunophenotypic characterization of MSCs isolated. Both manual and automated methods showed no difference in their phenotypic patterns.

A statistical analysis using Student’s t-test was conducted to assess potential differences between the two isolation methods. The results indicated no statistically significant differences (p > 0.05) in the expression levels of any of the analyzed markers, reinforcing the equivalence of both approaches in preserving the immunophenotypic characteristics of MSCs. The calculated t-values ranged between −1.02 and 0.89, further supporting the equivalence of both approaches in preserving the immunophenotypic characteristics of MSCs (Figure 2).

## Discussion

The optimization of MSCs isolation is crucial to ensure the viability and quality of cell therapy. It is essential to process the starting biological material with the highest possible yield, especially given the low proportion of MSCs within the total nucleated cells (0.01%–0.001%). Therefore, it is important to determine the method that best isolates MSCs with a higher proportion of colony-forming units.

In our study, we compared two isolation methods: manual and automated using the SEPAX system. We observed greater isolation of MNCs with the use of SEPAX, with a difference of 2.591 more cells compared to the manual method. These results are consistent with previous research conducted by Aktas et al. (2008) and Kaur et al. (2017) on bone marrow and umbilical cord samples, respectively.

However, when analyzing the yield in obtaining MSCs with both methods, no statistically significant differences were found. These findings suggest that greater efficiency in MNCs isolation does not necessarily correlate with better isolation of MSCs. Factors such as the bone marrow harvesting site, donor age, and underlying medical conditions may influence the quantity and quality of MSCs, affecting their therapeutic utility (Selle et al., 2022; Zaim et al., 2012). Although the Lin’s concordance correlation coefficient (rho_c) between the methods is high (0.849), indicating good overall concordance, this suggests that while SEPAX may show good overall concordance with the manual method in measuring levels, it does not seem to offer a significant improvement in MSCs isolation compared to the manual method in all cases.

When comparing the viability and characterization of MSCs isolated by both methods, no differences are observed in their capacity to differentiate into chondrogenic, adipogenic, and osteogenic lineages or in the phenotypic markers they exhibit. Additionally, when analyzing CFU-F and Ki67 marker expression, indicative of cell proliferation, no statistically significant differences are observed between MSCs isolated by both methods.

Regarding processing time, no significant differences were observed between the Sepax and manual methods for MNCs isolation. However, an economic evaluation revealed a substantial cost disparity: the total cost of the automatic Sepax system for processing a 100 mL bone marrow sample was 1,464.39€, whereas the manual method required only 851.99€. The primary cost difference stems from the single-use commercial kits required for the Sepax system (DGBS/Ficoll CS-900). This financial burden may limit the accessibility of automated systems, particularly in resource-limited settings.

Given these findings, optimizing MSC isolation protocols remains essential for clinical applications. Factors such as reproducibility, standardization, cell viability, infrastructure, and technical expertise should be carefully considered when implementing these methods in hospital settings or cell therapy units. While automation may offer advantages in standardization, the manual method proves to be a cost-effective, flexible, and viable alternative without compromising cell quality.

The optimization of MSC isolation protocols is a key aspect of their clinical application, especially considering the exponential growth that MSC-based therapies have experienced in recent decades. This is reflected in the increasing number of clinical trials registered on *clinicaltrials. gov*, which currently total 1,731, with 534 utilizing bone marrow-derived MSCs. This growth has driven the refinement of isolation and cell expansion strategies to optimize the viability and functionality of MSCs. Since these therapies are often targeted at conditions lacking effective treatments, it is crucial to maximize the quantity and quality of the harvested cells while ensuring their biological activity and regenerative properties. In this context, the implementation of efficient and standardized isolation methods becomes particularly relevant, as it directly impacts the efficacy and safety of MSC-based clinical applications (Rodríguez-Fuentes et al., 2021; Margiana et al., 2022).

In summary, our findings underscore the importance of critically evaluating MSCs isolation methods, considering not only efficiency in obtaining MNCs but also the ability to isolate high-quality MSCs for therapeutic applications. Although both methods produce functionally equivalent MSCs, economic and practical considerations must be weighed, particularly for large-scale or clinical-grade cell production. Future studies should focus on refining manual processing techniques to enhance reproducibility while maintaining their economic advantage, ensuring a balance between cost-effectiveness, standardization, and clinical applicability for the successful translation of MSC-based therapies into clinical practice.

## Data Availability

The original contributions presented in the study are included in the article/supplementary material, further inquiries can be directed to the corresponding author.

## References

[B1] Aktas M. Buchheiser A. Houben A. Reimann V. Radke T. Jeltsch K. (2010). Good manufacturing practice-grade production of unrestricted somatic stem cell from fresh cord blood. Cytotherapy 12 (3), 338–348. 10.3109/14653241003695034 20370349

[B2] Aktas M. Radke T. F. Strauer B. E. Wernet P. Kogler G. (2008). Separation of adult bone marrow mononuclear cells using the automated closed separation system Sepax. Cytotherapy 10 (2), 203–211. 10.1080/14653240701851324 18368599

[B3] Chu D. T. Phuong T. N. T. Tien N. L. B. Tran D. K. Thanh V. V. Quang T. L. (2020). An update on the progress of isolation, culture, storage, and clinical application of human bone marrow mesenchymal stem/stromal cells. Int. J. Mol. Sci. 21 (3), 708. 10.3390/ijms21030708 31973182 PMC7037097

[B4] Dominici M. Le Blanc K. Mueller I. Slaper-Cortenbach I. Marini F. Krause D. (2006). Minimal criteria for defining multipotent mesenchymal stromal cells. The International Society for Cellular Therapy position statement. Cytotherapy 8 (4), 315–317. 10.1080/14653240600855905 16923606

[B5] Gudleviciene Z. Kundrotas G. Liudkeviciene R. Rascon J. Jurga M. (2014). Quick and effective method of bone marrow mesenchymal stem cell extraction. Open Med. Wars. Pol. 10 (1), 44–49. 10.1515/med-2015-0008 PMC5152963 28352676

[B6] Kaur I. Zulovich J. M. Gonzalez M. McGee K. M. Ponweera N. Thandi D. (2017). Comparison of two methodologies for the enrichment of mononuclear cells from thawed cord blood products: the automated Sepax system versus the manual Ficoll method. Cytotherapy 19 (3), 433–439. 10.1016/j.jcyt.2016.11.010 28034522

[B7] Margiana R. Markov A. Zekiy A. O. Hamza M. U. Al-Dabbagh K. A. Al-Zubaidi S. H. (2022). Clinical application of mesenchymal stem cell in regenerative medicine: a narrative review. Stem cell Res. and Ther. 13 (1), 366. 10.1186/s13287-022-03054-0 35902958 PMC9330677

[B8] Najar M. Raicevic G. Fayyad-Kazan H. Bron D. Toungouz M. Lagneaux L. (2016). Mesenchymal stromal cells and immunomodulation: a gathering of regulatory immune cells. Cytotherapy 18 (2), 160–171. 10.1016/j.jcyt.2015.10.011 26794710

[B9] Nasef A. Fouillard L. El-Taguri A. Lopez M. (2007). Human bone marrow-derived mesenchymal stem cells. Libyan J. Med. 2 (4), 190–201. 10.4176/070705 21503244 PMC3078252

[B10] Rodríguez-Fuentes D. E. Fernández-Garza L. E. Samia-Meza J. A. Barrera-Barrera S. A. Caplan A. I. Barrera-Saldaña H. A. (2021). Mesenchymal stem cells current clinical applications: a systematic review. Archives Med. Res. 52 (1), 93–101. 10.1016/j.arcmed.2020.08.006 32977984

[B11] Selle M. Koch J. D. Ongsiek A. Ulbrich L. Ye W. Jiang Z. (2022). Influence of age on stem cells depends on the sex of the bone marrow donor. J. Cell. Mol. Med. 26 (5), 1594–1605. 10.1111/jcmm.17201 35088539 PMC8899192

[B12] Thirumala S. Goebel W. S. Woods E. J. (2009). Clinical grade adult stem cell banking. Organogenesis 5 (3), 143–154. 10.4161/org.5.3.9811 20046678 PMC2781095

[B13] Zaim M. Karaman S. Cetin G. Isik S. (2012). Donor age and long-term culture affect differentiation and proliferation of human bone marrow mesenchymal stem cells. Ann. Hematol. 91 (8), 1175–1186. 10.1007/s00277-012-1438-x 22395436

